# Evaluation of the bacterial ocular surface microbiome in clinically normal cats before and after treatment with topical erythromycin

**DOI:** 10.1371/journal.pone.0223859

**Published:** 2019-10-11

**Authors:** Joshua E. Darden, Erin M. Scott, Carolyn Arnold, Elizabeth M. Scallan, Bradley T. Simon, Jan S. Suchodolski

**Affiliations:** 1 Department of Small Animal Clinical Sciences, College of Veterinary Medicine & Biomedical Sciences, Texas A&M University, College Station, Texas, United States of America; 2 Department of Large Animal Clinical Sciences, College of Veterinary Medicine & Biomedical Sciences, Texas A&M University, College Station, Texas, United States of America; Western University of Health Sciences, United States of America

## Abstract

The ocular surface microbiome of veterinary species has not been thoroughly characterized using next generation sequencing. Furthermore, alterations in the feline ocular surface microbiome over time or following topical antibiotic treatment are unknown. Aims of this study were to further characterize the ocular surface microbiome of healthy cats and to identify whether there are microbial community changes over time and following topical antibiotic use. Twenty-four eyes from twelve adult, research-bred, female spayed domestic shorthaired cats were evaluated. Erythromycin ophthalmic ointment (0.5%) was applied to the ocular surface of one randomly assigned eye per cat three times daily for 7 days, while the fellow eye served as an untreated control. The ocular surface was sampled by swabbing the inferior conjunctival fornix of both eyes prior to initiating treatment (day 0), after 1 week of treatment (day 7), and 4 weeks after concluding treatment (day 35). Genomic DNA was extracted from the swabs and sequenced using primers that target the V4 region of bacterial 16S rRNA genes. At baseline, the most common bacterial phyla identified were Proteobacteria (42.4%), Firmicutes (30.0%), Actinobacteria (15.6%), and Bacteroidetes (8.1%). The most abundant bacterial families sequenced were Corynebacteriaceae (7.8%), Helicobacteraceae (7.5%), Moraxellaceae (6.1%), and Comamonadaceae (5.6%). Alpha and beta diversity measurements were largely unchanged in both treatment and control eyes over time. However, univariate and linear discriminant analyses revealed significant and similar changes in the abundance of some bacterial taxa over time in both treatment and control eyes. Overall, the feline ocular surface microbiome remained stable over time and following topical antibiotic therapy.

## Introduction

The ocular surface consists of the corneal epithelium along with the most prominently exposed mucous membrane of the body, the conjunctiva. Furthermore, it represents a predominately open system that is subject to a barrage of pathogenic and non-pathogenic organisms as it is constantly in contact with its environment [1–3]. This open exposure relies on adaptive and innate immunologic constructs to prevent pathogenic colonization of the ocular surface [4, 5]. However, there likely is a component of harmonious interaction between microbiota acting in a commensal and symbiotic nature against overgrowth or colonization of pathogenic microorganisms [2, 6–17]. This mucosal tolerance allows normal ocular surface microbiota to occupy its environment yet remain non-stimulatory [5].

Infectious conjunctivitis in cats is associated with viral or bacterial etiologies and is a frequent cause of ocular problems in veterinary medicine, where both pathogenic and opportunistic bacterial microorganisms contribute to the disease process [1,18–21]. Standard of care for the treatment of feline conjunctivitis and keratitis consists of topical ophthalmic antibiotics, such as erythromycin [1–3,22]. Antibiotics may negatively alter the microbial community of the ocular surface [11], potentially contributing to opportunistic invasion of pathogenic species and ocular disease [12, 13].

The microorganisms inhabiting the ocular surface of veterinary species have been evaluated traditionally using standard culture-based techniques from corneal and conjunctival swabs [1–3, 18–19, 21–35]. Additionally, biochemical tests and mass spectrometry have been utilized to identify microbes that were cultivated through the aforementioned techniques [1–3, 19, 23–27]. The percent of total positive cultures from healthy feline eyes is low, with gram positive bacteria such as *Staphylococcus*, *Streptococcus*, and *Corynebacterium* spp. representing the most commonly cultivated microorganisms [1–3, 18–22]. Due to the inability of several studies to culture bacteria from the eyes of healthy cats [2, 3, 21], the ocular surface was speculated to be sterile [2, 19, 22]. It is now known that limitations exist for standard culture-based techniques as many bacteria are not easily cultivable [36]. With this shortcoming of accurately depicting the complete bacterial composition of an environment, terminology describing the once mysterious population of microbiota has been erected giving rise to the phrase the “uncultured microbial majority” [36]. To identify and characterize this enigmatic population of microorganisms, molecular-based methods that target specific DNA markers allow microbes to be defined by their genomes, giving rise to the microbiome [6–10, 13, 37–41].

The arrival of molecular-based methods, such as 16S rRNA gene sequencing, has allowed in-depth and detailed species identification of the bacterial microbiota residing on the ocular surface in humans [6, 8, 12, 13, 37–40, 42, 43], while limited preliminary studies have been published in veterinary species such as cats [41], horses [44], and dogs [45]. Presently, there are no published studies evaluating the ocular surface microbiome of cats using molecular-based techniques that evaluate temporal stability and the impact of antibiotic usage. Knowledge of these microbial populations, how to sample them, and how they change over time and with treatment may one day lead to an improved understanding of ocular diseases in both veterinary and physician ophthalmology. This study was designed to examine the ocular surface microbiome of healthy cats using next-generation sequencing. The aims of the study were, 1) to further describe the resident ocular microbiota in healthy research-bred cats at baseline, 2) to assess the temporal stability the ocular microbiota at three distinct time points, and 3) to evaluate the influence of antimicrobial therapy before, immediately after 1 week of therapy, and after 1-month hiatus of antimicrobial use.

## Materials and methods

### Participants

The study was approved by the Texas A&M University Institutional Animal Care and Use Committee (Animal Use Protocol #2017–0313) and performed in accordance with the Association for Research in Vision and Ophthalmology Statement on the Use of Animals in Ophthalmic and Vision Research (https://www.arvo.org/About/policies/statement-for-the-use-of-animals-in-ophthalmic-and-vision-research/). Twelve healthy research-bred (Liberty Research Inc., Waverly, NY) adult domestic shorthair cats were included in the study. All twelve cats were spayed females with ages ranging from 1 to 1.5 years old. Cats were housed together in groups of 2 to 4 in adjacent free-ranging enclosures of 2.4 m L x 2.4 m W x 3.0 m H. The facility provided controlled light cycle (12/12-hour light/dark), temperature (21–22° C) and humidity (55–60%) conditions. Environmental enrichment consisted of toys, scratch posts, raised condos, and bedding. One wall of each enclosure consisted of large windows that provided natural light. Cats were well accustomed to handling and acclimated to ophthalmic examinations for two weeks before the study was initiated. Water and food were available at all times.

### Sample collection

All cats had a complete ophthalmic examination performed by a board-certified veterinary ophthalmologist (EMS) and ophthalmology resident in training (JED) to ensure they were free of ocular disease. As described in detail previously [44], this included evaluation of the anterior segment of the eye by slit-lamp biomicroscopy (SL-17, Kowa Optimed Inc., Torrance, CA), and the posterior segment of the eye by indirect ophthalmoscopy (Vantage Plus Wireless Headset, Keeler Instruments Inc., Malvern, PA). A routine minimal ophthalmic database that included fluorescein staining (Amcon Laboratories Inc., St. Louis, MO) and tonometry (TonoVet, Icare VET, Jorgensen Laboratories Inc., Loveland, CO) was performed.

Baseline conjunctival samples were collected before fluorescein staining in order to prevent contamination or dilution of the sample. Sample collection was performed in awake cats using gentle manual restraint following the application of one drop 0.5% proparacaine (Bausch & Lomb Inc., Bridgewater, NJ) to the ocular surface of each eye to provide topical analgesia. As described previously [44], swabs of the inferior conjunctival fornix were collected from both eyes of every cat at three separate time points. Two Isohelix buccal swabs (Boca Scientific Inc., Westwood, MA) were used per eye and each side of the swab was rubbed in the inferior conjunctival fornix 10 times. Swabs were immediately transferred into DNeasy PowerBead tubes with 750-μl buffer containing guanidine thiocyanate (QIAGEN Inc., Germantown, MD). To control for environmental contamination, an unused swab containing one drop of 0.5% proparacaine was collected at the same time and place as the conjunctival swabs. All samples were immediately stored for no longer than 24 hours at 4° C until the extractions were performed.

Once baseline samples (day 0) were collected, one eye of each cat was randomly selected for treatment with a topical broad-spectrum antibiotic ointment, 0.5% erythromycin (Bausch & Lomb Inc., Bridgewater, NJ), commonly used for the treatment of feline conjunctivitis and ulcerative keratitis. Utilizing online software (https://www.randomizer.org), randomization of eyes into treatment and control groups for each cat was established. One quarter-inch strip of erythromycin ophthalmic ointment was applied directly to the ocular surface of the randomly selected eye of each cat three times daily for 7 days, while the fellow eye served as an untreated control. Care was taken to avoid contact of the ointment with the outer surface of the eyelids or periocular skin, and handlers wore nitrile gloves while administering the ophthalmic medication. Repeat inferior conjunctival fornix swabs were collected on day 7 (after completion of antibiotic therapy) and day 35 (one month after antibiotic therapy ended).

### DNA extraction and sequencing

Genomic DNA was extracted from the conjunctival swabs and negative controls using a single 100 tube DNeasy Powersoil DNA isolation kit (Cat ID 12888–100, Lot number 157049640, QIAGEN, Inc., Germantown, MD) following the manufacturer’s instructions. Negative controls consisting of one unused swab and one drop 0.5% proparacaine were collected at each timepoint. All three negative controls did not show amplification on PCR and, therefore, were not sequenced with the conjunctival swabs. Sequencing of the 16S rRNA gene V4 variable region was performed at MR DNA Laboratory (www.mrdnalab.com, Shallowater, TX, USA) on an Illumina MiSeq platform (Illumina Inc., San Diego, CA) to produce 2x300 paired-end reads using 515F (5’ -GTGYCAGCMGCCGCGGTAA- 3’) and 806R (5´-GGACTACNVGGGTWTCTAAT- 3´) primers, as described previously [44].

### Data analysis

Statistical analysis was performed as previously described [44]. Sequences were processed and analyzed using Quantitative Insights Into Microbial Ecology (QIIME 2) [46]. Raw sequence data were de-multiplexed and low-quality reads were filtered using default parameters for QIIME. Chimeric sequences were detected and removed using DADA2 prior to analysis [47]. Operational taxonomic units (OTUs) were assigned and clustered using an open-reference protocol in QIIME and defined as having at least 97% similarity against the Greengenes reference database [48,49]. For downstream analysis, unassigned contaminant sequences and those assigned as mitochondria, chloroplasts, or the phylum Cyanobacterium, were excluded from further analysis. Data were deposited in the National Center for Biotechnology Information (NCBI) Sequence Read Archive (SRA) under the accession number SRP161480.

Alpha diversity metrics (observed OTUs, Shannon, and Chao1) were used to compare species richness and evenness between eyes at baseline and among control and treatment eyes over time. Data were assumed to follow a non-normal distribution. Therefore, a non-parametric Wilcoxon matched-pairs signed-ranks test was used for statistical comparison between treatment and control eyes at baseline. A non-parametric Friedman test, followed by a Dunn’s multiple comparison post-test were performed to assess differences in treatment and control eyes over three time points [49]. Statistical analysis was performed using the software package PRISM (PRISM 7, GraphPad Software Inc., San Diego, CA).

Beta diversity, which assesses bacterial community composition, was determined using both weighted and unweighted UniFrac metrics to measure similarity between samples, and evaluated for clustering with Principle Coordinate Analysis (PCoA) plots. An Analysis of Similarity test (ANOSIM) within PRIMER 6 (PRIMER-E Ltd. Luton, UK) was used to assess differences in bacterial community composition between samples.

Differences in the relative abundance of bacterial taxa between eyes at baseline, and among control and treatment eyes over time, were examined. Most datasets did not meet the assumption of normality using the Shapiro-Wilk test (JMP Pro 14, SAS, Marlow, Buckinghamshire). Therefore, a non-parametric Mann-Whitney U test was used to compare treatment and control eyes at baseline. A non-parametric Friedman test was applied to assess differences in treatment and control eyes over three time points (PRISM 7, GraphPad Software Inc., San Diego, CA). A Dunn’s multiple comparison post-test was then used to determine which time points were significantly different. P-values were adjusted for multiple comparisons and corrected for false discovery rate [50]. P- and q-values <0.05 were considered statistically significant.

To analyze the abundance of bacterial taxa in treatment and control eyes and their associations with each time point, linear discriminant analysis effect size (LEfSe) was performed using Calypso [51, 52].

## Results

### Sequence analysis

Initial DNA quantities extracted from the conjunctival swabs are reported in S1 Table. All sequences were rarified to an even sequencing depth of 15,999 sequences per sample to correct for unevenness between samples. A total of 72 samples were collected from 24 eyes at three time points, and 3,350,060 sequences were amplified (min: 15,999, max: 87,824, median: 45,005, mean: 46,528, standard deviation: 16,666). For each individual sample, the relative abundance of bacteria was defined.

### Healthy feline eyes at baseline

#### Species richness and diversity

**Fig 1:**
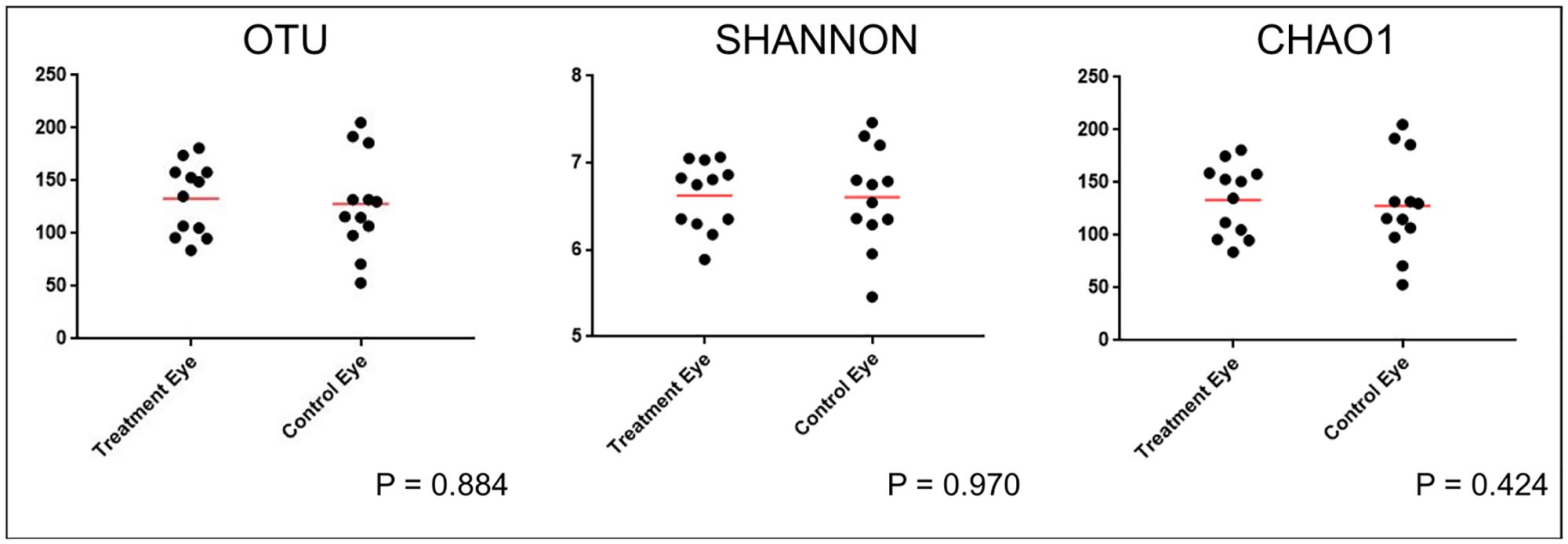
Scatter plots of 16S rRNA gene sequences obtained from 12 healthy cats (24 eyes), comparing treatment and control groups at baseline (day 0). Each dot represents one eye. There is no difference in alpha diversity between eyes at baseline (Wilcoxon match-pairs signed-ranks test).

Baseline samples from treatment and control eyes were compared prior to antibiotic treatment on Day 0 (S2 Table). Three alpha diversity metrics were analyzed including observed OTUs, Shannon, and Chao1 to examine taxonomic diversity within a sample. Wilcoxon match-pairs signed-ranks test revealed no difference in alpha diversity between control eyes and treatment eyes at baseline. Thus, there was no difference in species richness, evenness, or abundance between eyes at baseline (Fig 1).

#### Microbial community structure

**Fig 2:**
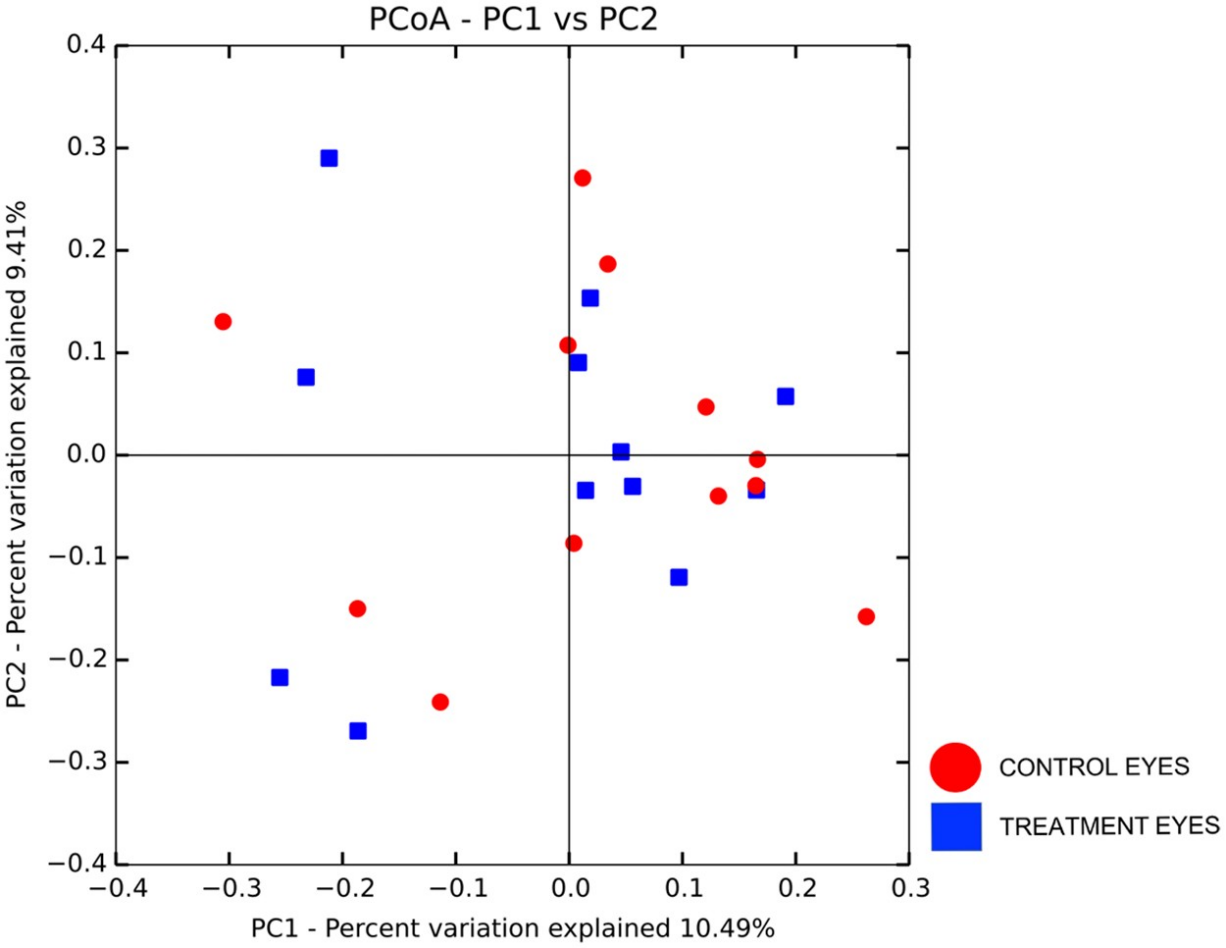
Principle coordinate analysis plot (PCoA) of unweighted UniFrac distance matrices between treatment and control eyes at baseline (day 0). Each dot represents the microbial composition of one eye. Clustering was not observed indicating no difference in beta diversity between eyes at baseline.

Two beta diversity metrics, weighted UniFrac and unweighted UniFrac, were analyzed to examine taxonomic diversity between samples. There was no difference in community structure between treatment and control eyes at baseline (R = -0.037, R = -0.052, respectively, p > 0.05). Treatment eyes did not cluster differently from control eyes at baseline (Fig 2).

#### Microbial community composition

**Fig 3:**
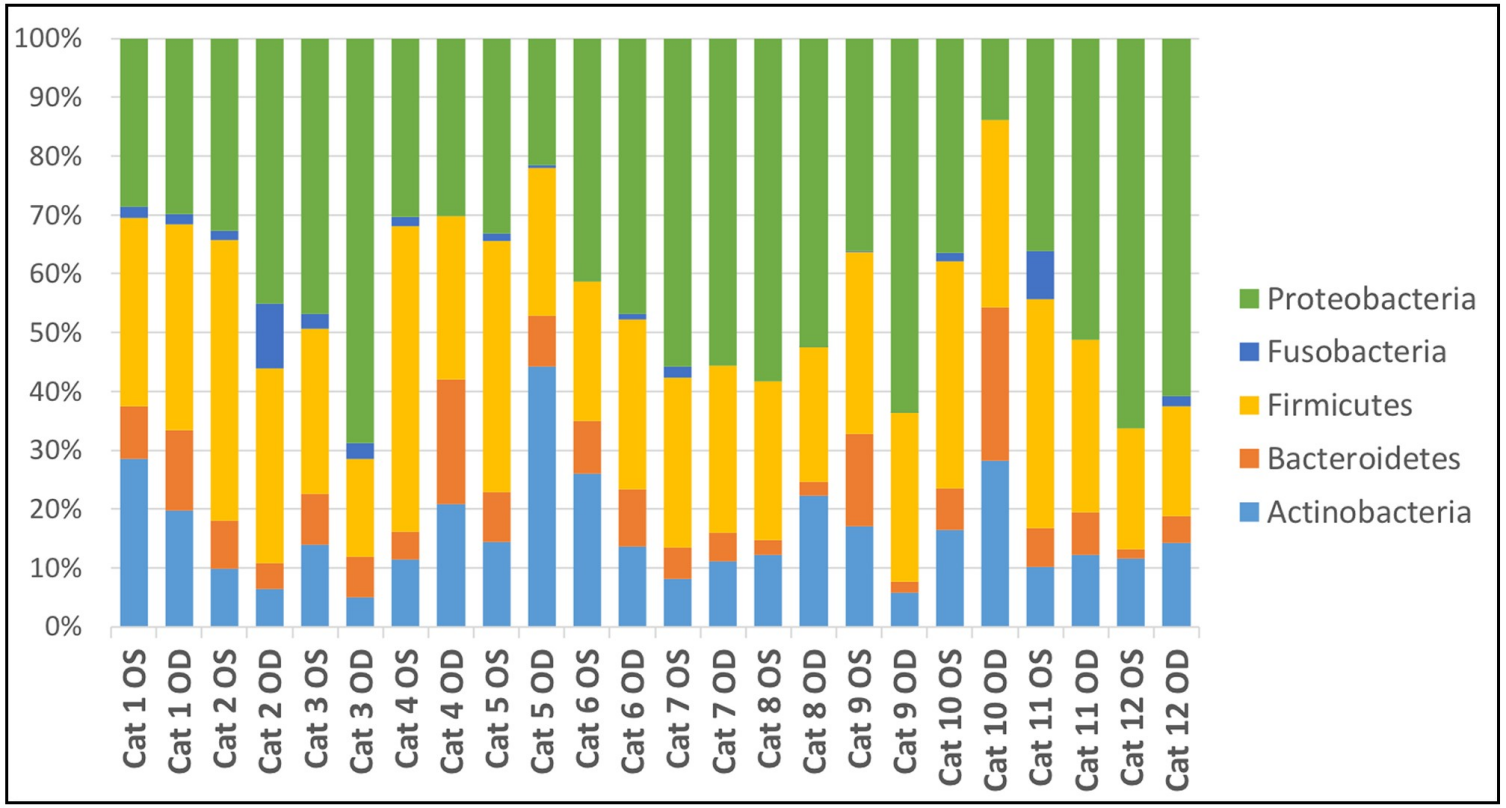
Ocular surface microbiome composition in healthy cats. Relative abundance of taxa present at >1% and annotated to the level of bacterial phylum at baseline (day 0). Each bar chart represents the left (OS) or right (OD) eyes of 12 cats.

Bacterial taxa abundance did not differ between treatment and control eyes at baseline. Data from all 24 eyes were averaged to describe the bacterial taxa composition of the healthy feline ocular surface. A total of 5 bacterial phyla were detected and 4 phyla were present in all 24 eyes (Table 1). The most common phyla were Proteobacteria (42.4%), followed by Firmicutes (30.0%), Actinobacteria (15.6%), and Bacteroidetes (8.1%) (Fig 3).

**Table 1:** Taxa present at ≥1% mean relative abundance in healthy cats. Mean percentages and standard deviation of bacteria present at baseline annotated to the level of phylum, family, and genus, based on sequencing of 16S rRNA genes.

Taxon	Healthy Cats at Baseline		
Phylum Family -*Genus*	Mean %	SD %	Number of eyes with positive detection (n = 24)
**Proteobacteria**	42.4	14.4	24
Helicobacteraceae	7.5	1.4	23
-*Unclassified Helicobacteraceae*	7.5	1.4	20
Moraxellaceae	6.1	3.5	23
*-Acinetobacter spp*.	4.9	3.1	23
Comamonadaceae	5.6	4.4	23
-*Unclassified Comamonadaceae*	1.9	1.8	18
*-Delftia spp*.	1.6	2.1	14
Pseudomonadaceae	5.4	3.7	24
*-Pseudomonas spp*.	4.7	3.3	24
Pasteurellaceae	3.3	3.7	19
-*Unclassified Pasteurellaceae*	2.8	3.0	17
Halomonadaceae	1.7	1.9	22
*-Halomonas spp*.	1.7	1.9	22
Enterobacteriaceae	1.8	1.9	17
-*Unclassified Enterobacteriaceae*	1.4	1.6	16
Neisseriaceae	1.4	1.6	17
-*Unclassified Neisseriaceae*	1.0	1.0	10
Xanthomonadaceae	1.4	2.9	14
Rhodobacteraceae	1.0	1.3	13
Oxalobacteraceae	1.0	1.0	13
Sphingomonadaceae	1.0	1.0	11
**Firmicutes**	30.0	8.3	24
Staphylococcaceae	5.3	5.2	24
*-Staphylococcus spp*.	4.8	5.3	23
Streptococcaceae	4.4	2.6	24
*-Streptococcus spp*.	4.3	2.7	24
Bacillaceae	4.4	4.5	18
*-Bacillus spp*.	3.0	3.4	16
*-Anoxybacillus spp*.	1.0	1.9	6
*-Geobacillus spp*.	1.0	1.4	7
Aerococcaceae	2.5	2.1	21
-*Unclassified Aerococcaceae*	1.7	2.0	20
Ruminococcaceae	1.7	2.3	15
-*Unclassified Ruminococcaceae*	1.0	1.3	12
Tissierellaceae	1.4	4.6	11
*-Anaerococcus spp*	1.1	4.6	6
Lactobacillaceae	1.1	2.1	10
*-Lactobacillus spp*.	1.2	2.3	10
Enterococcaceae	1.1	1.9	10
-*Unclassified Enterococcaceae*	1.0	1.8	8
Exiguobacteraceae	1.1	1.3	15
-*Unclassified Exiguobacteraceae*	1.0	1.3	13
Planococcaceae	1.0	1.5	11
-*Unclassified Planococcaceae*	1.0	1.3	9
Gemellaceae	1.0	1.5	11
-*Unclassified Gemellaceae*	1.0	1.5	11
Lachnospiraceae	1.0	1.4	12
Erysipelotrichaceae	1.0	1.1	14
Clostridiaceae	1.0	1.0	12
**Actinobacteria**	15.6	8.8	24
Corynebacteriaceae	7.8	6.7	23
*-Corynebacterium spp*.	7.8	6.7	23
Microbacteriaceae	2.2	2.2	18
-*Unclassified Microbacteriaceae*	2.0	2.1	16
Micrococcaceae	1.9	1.9	19
*-Micrococcus spp*.	1.0	1.3	12
Bifidobacteriaceae	1.0	1.1	13
*-Bifidobacterium spp*.	1.0	1.1	13
**Bacteroidetes**	8.1	5.7	24
Weeksellaceae	4.7	5.7	20
*-Cloacibacterium spp*.	4.4	5.6	19
Bacteroidaceae	1.0	1.0	11
*-Bacteroides spp*.	1.0	1.0	11
Porphyromonadaceae	1.0	1.0	18
*-Porphyromonas spp*.	1.0	1.0	15
**Fusobacteria**	**1.6**	**2.6**	**17**
Fusobacteriaceae	1.2	2.1	14
*-Fusobacterium spp*.	1.2	2.0	14

**Fig 4:**
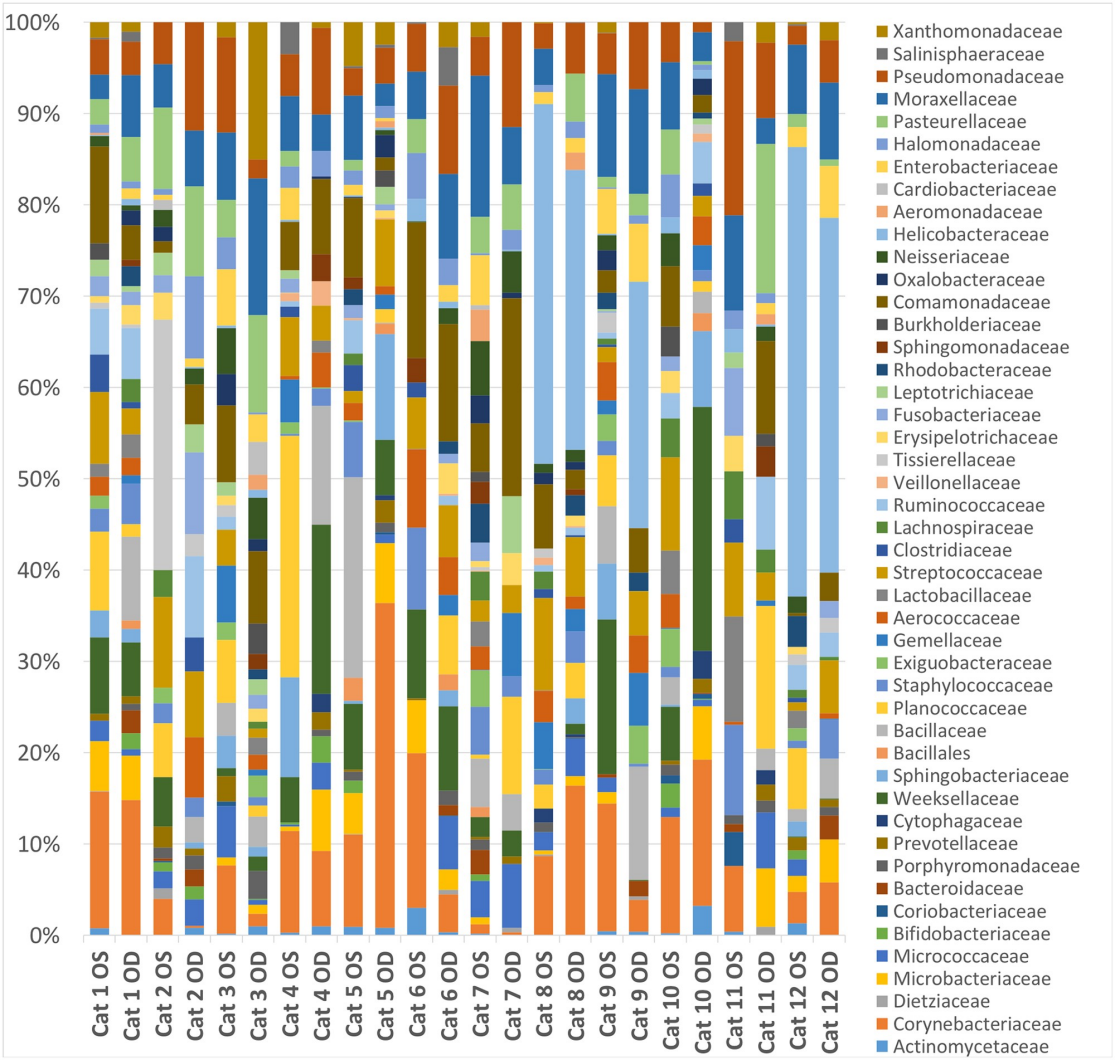
Ocular surface microbiome composition in healthy cats. Relative abundance of taxa present at >1% and annotated to the level of bacterial family at baseline (day 0). Each bar chart represents the left (OS) or right (OD) eyes of 12 cats.

Thirty-four bacterial families at >1% relative abundance were detected and three families were present in all 24 eyes (Table 1). The most common bacterial families sequenced were Corynebacteriaceae (7.8%), Helicobacteraceae (7.5%), Moraxellaceae (6.1%), and Comamonadaceae (5.6%). Other commonly identified families present in most eyes included Pseudomonadaceae (5.4%), Staphylococcaceae (5.3%), and Weeksellaceae (4.7%) (Fig 4). Streptococcaceae, Bacillacea, and Micrococcaceae represented 4.4%, 4.4%, and 1.9% of the bacterial families sequenced, respectively. The relative abundances of bacterial taxa varied both between eyes and between cats; however, the overall composition remained consistent (Figs 3 and 4). An average of 353 different OTUs were detected throughout all samples.

### Temporal variability of ocular surface microbiome in control eyes

Two additional samples were collected from control eyes one week (day 7) and five weeks (day 35) after the baseline collection (day 0), in order to investigate the temporal stability of the ocular surface microbiome in healthy cats.

#### Species richness and diversity

**Fig 5:**
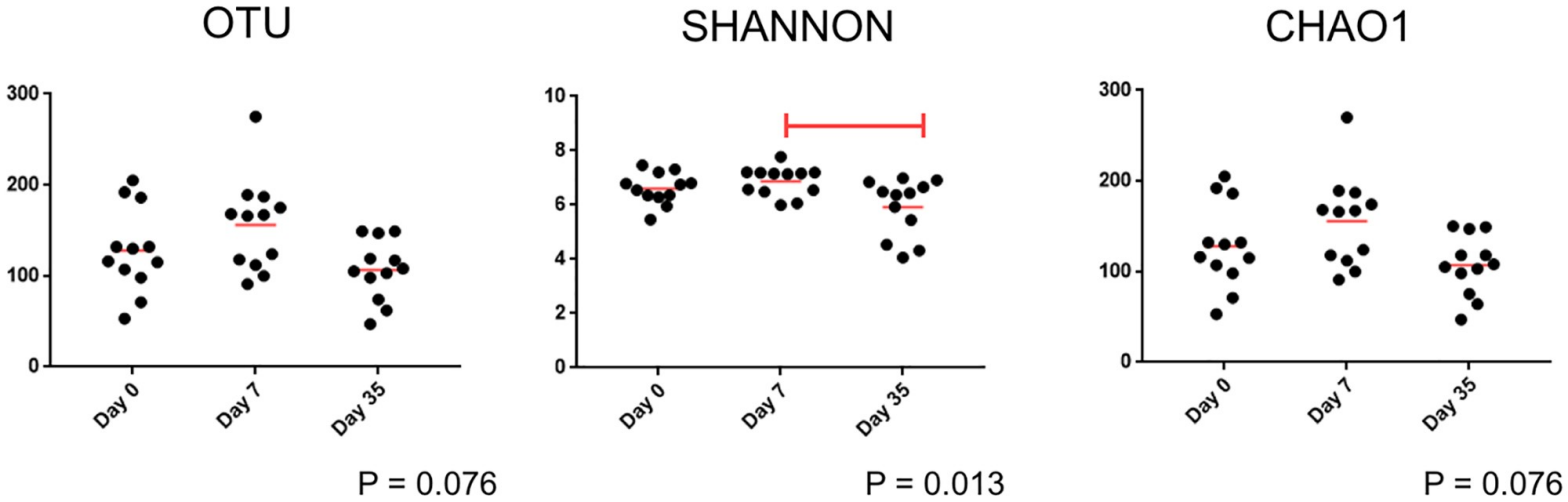
Scatter plots of 16S rRNA gene sequences obtained from 12 control eyes of 12 healthy cats at 3 time points: Day 0, day 7, day 35. There is no difference in observed OTUs and Chao1 in control eyes over time (Friedman test and Dunn’s post-test). Shannon diversity, which evaluates species richness and evenness, is lower on day 35 compared to day 7.

Alpha diversity was largely unchanged in control eyes over time (S3 Table and Fig 5). There was no difference in observed OTUs and Chao1 based on the sampling time point. However, Shannon diversity was lower on day 35 compared to day 7 (p = 0.013).

#### Microbial community structure

**Fig 6:**
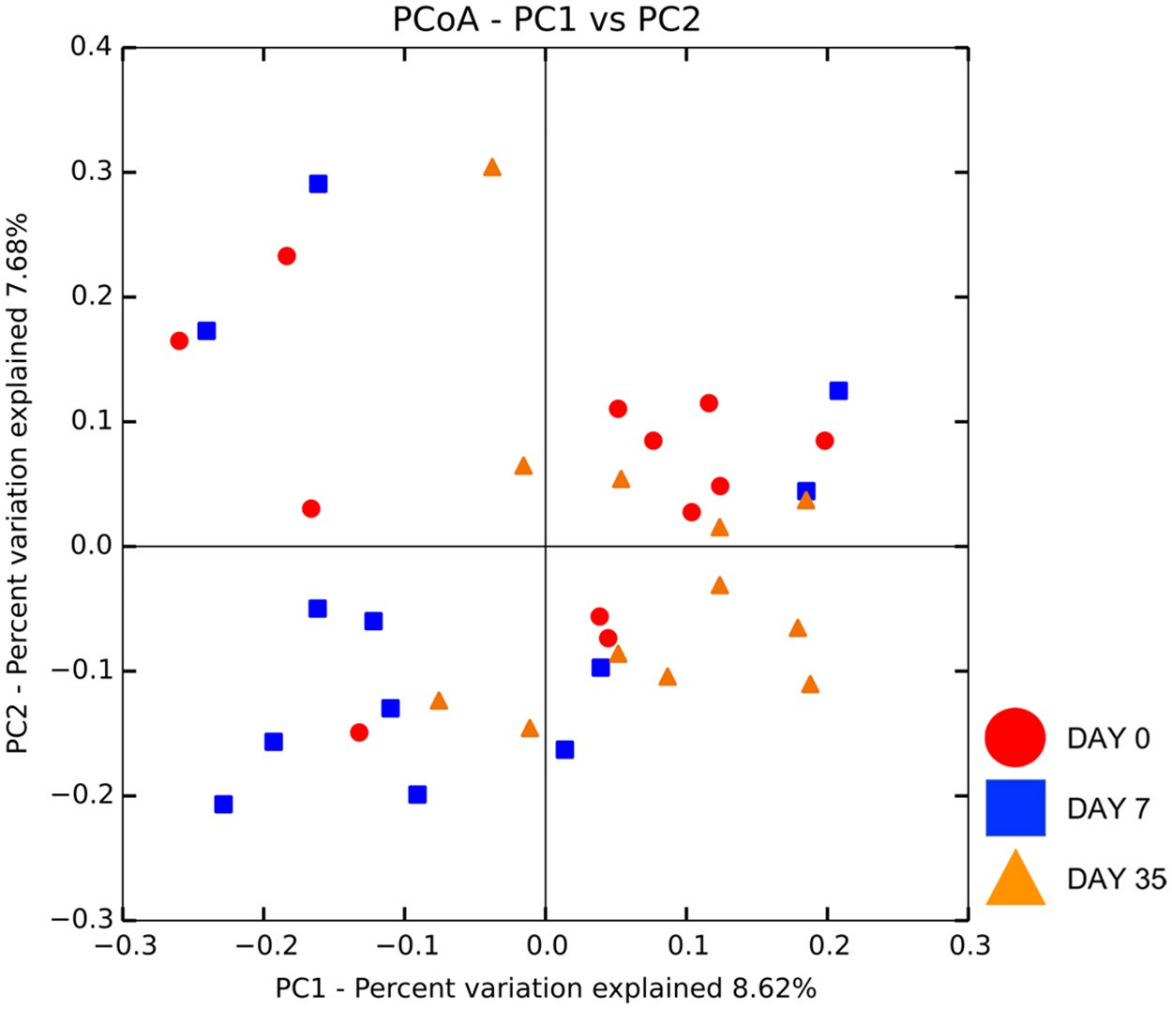
Principle coordinate analysis plot (PCoA) of unweighted UniFrac distance matrices of 12 control eyes from 12 healthy cats at three time points: Day 0, day 7, day 35. Clustering was not observed indicating no difference in beta diversity in control eyes over time.

Beta diversity did not differ in control eyes sampled over time as visible by the lack of clustering in the PCoA plot (Fig 6). No difference in microbial communities was detected with ANOSIM (unweighted UniFrac, R = 0.088, R = 0.109, R = 0.125 for day 0 vs. 7, day 0 vs. 35, and day 7 vs. 35, respectively, p > 0.05); (weighted UniFrac, R = 0.085, R = 0.262, R = 0.159 for day 0 vs. 7, day 0 vs. 35, and day 7 vs. 35, respectively, p > 0.05).

#### Microbial community composition

**Fig 7:**
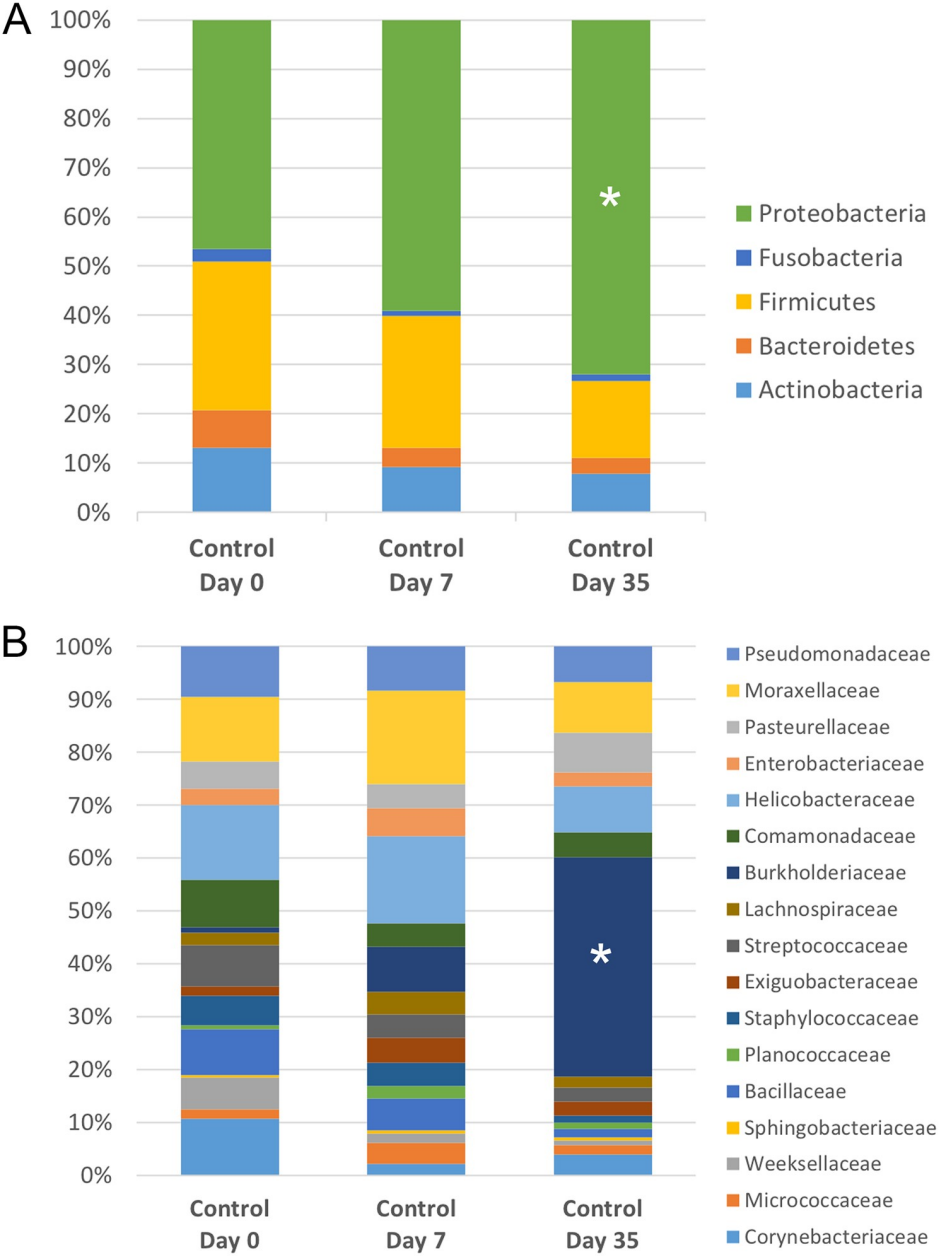
Temporal composition by bacterial phyla (A) and families (B) in control eyes. Bars represent mean percentage of taxa present at ≥ 3% mean relative abundance. **(A)** Note the relative abundance of Proteobacteria (*) is increased on day 35, and **(B)** the relative abundance of Burkholderiacaea (*) is increased on day 35.

The relative abundance of bacteria in control eyes sampled over time is illustrated in Fig 7. Four taxa were differentially abundant on the ocular surface of control eyes at each timepoint (Table 2). At the phylum level, Proteobacteria were increased on day 35 compared to day 0 and day 7 (p < 0.001, q = 0.001). At the genus level, *Burkholderia* spp. were enriched in control eyes on day 35 compared to baseline (day 0) and day 7 (p < 0.001, q = 0.004).

**Table 2:** Temporal variation of bacterial taxa isolated from the ocular surface of control eyes of healthy cats at three time points. Median relative percentages and ranges of bacterial groups, annotated to level of phylum, family and genus, based on sequencing of 16S rRNA genes.

Taxa	Day 0		Day 7		Day 35			
Phylum Family *-Genus*	Median %	Range %	Median %	Range %	Median %	Range %	p-value*	q-value**
**Proteobacteria**	42.1^a^	28.9–67.8	58.5^a^	41.5–79.4	64.1^b^	48.2–93.5	**<0.001**	**0.001**
Burkholderiaceae	0^a^	0–3.1	0.8^a^	0–28.3	3.7^b^	0.8–86.5	**<0.001**	**0.005**
*-Burkholderia*	0^a^	0–3.1	0.8^a^	0–28.3	3.7^b^	0.8–86.4	**<0.001**	**0.004**
**Firmicutes**	28.3^a^	16.2–42.6	26.7^a^	2–43.8	13.9^b^	4.9–32.5	**0.018**	0.055
Staphylococcaceae	2.8^a^	0.3–8.9	2.5^a^	0.1–9.9	0.4^b^	0–4	**0.005**	0.080
*-Staphylococcus*	2.0^a^	0–8.9	2.3^a,b^	0.1–9.9	0.4^b^	0–4	**0.026**	0.131
**Actinobacteria**	12.6^a^	4.5–24.1	8.6^a,b^	2–15.1	5.6^b^	0.5–31.3	0.050	0.075
Corynebacteriaceae	7^a^	0.2–14.2	0.5^b^	0–5.7	1.4^a,b^	0–20.6	**0.003**	0.074
*-Corynebacterium*	7^a^	0.2–14.2	0.5^b^	0–5.7	1.4^a,b^	0–20.6	**0.003**	0.070
**Bacteroidetes**	6.6^a^	2–20.7	3.8^a,b^	0.5–6.4	2.0^b^	0.4–11.4	**0.028**	0.056

Median values not sharing a common superscript differ significantly (p < 0.05, Dunn’s multiple comparison post-test).

*: P-values based on the Friedman test

**: Q-values adjusted based on the Benjamini & Hochberg False discovery rate

Based on LEfSe analysis, the relative abundance of several bacterial taxa were altered over time (Table 3). As noted with the analysis of bacterial groups using univariate testing (Friedman and Dunn’s multiple comparison tests), *Burkholderia* spp. and their associated family, Burkholderiacaea, and phylum, Proteobacteria, were amplified on day 35 among control eyes (Tables 2 and 3).

**Table 3:** Linear discriminant analysis of bacterial taxa with LDA scores > 3.0 in control eyes and their associations with each time point.

Taxa	LDA	Time point
Phylum Family *-Genus*		
**Bacteroidetes**	4.87	Day 0
**Firmicutes**	4.96	Day 0
**Proteobacteria**	5.15	Day 35
Gemellaceae	3.89	Day 0
Bacteroidaceae	3.90	Day 0
Bifidobacteriaceae	4.01	Day 0
Staphylococcaceae	4.15	Day 0
Corynebacteriaceae	4.38	Day 0
Dietziaceae	3.75	Day 7
Succinivibrionaceae	3.84	Day 7
Cardiobacteriaceae	3.91	Day 7
Planococcaceae	3.93	Day 7
Enterobacteriaceae	3.94	Day 7
Micrococcaceae	4.00	Day 7
Campylobacteraceae	4.03	Day 7
Flavobacteriaceae	4.24	Day 7
Porphyromonadaceae	3.93	Day 35
Burkholderiaceae	5.17	Day 35
-*Unclassified Bradyrhizobiaceae*	3.61	Day 0
*-Bifidobacterium*	3.63	Day 0
-*Unclassified Gemellaceae*	3.68	Day 0
*-Bacteroides*	3.73	Day 0
*-Corynebacterium*	4.40	Day 0
*-Dietzia*	3.30	Day 0
*-Arthrobacter*	3.31	Day 7
*-Capnocytophaga*	3.33	Day 7
*-Roseburia*	3.43	Day 7
*-Dorea*	3.45	Day 7
*-Anaerococcus*	3.47	Day 7
*-Campylobacter*	3.48	Day 7
*-Sphingomonas*	3.60	Day 7
*-Dialister*	3.75	Day 7
-*Unclassified Nocardioidaceae*	3.75	Day 7
-*Unclassified Enterobacteriaceae*	4.01	Day 7
*-Acinetobacter*	4.50	Day 7
*-Enterococcus*	3.60	Day 35
*-Porphyromonas*	3.77	Day 35
*-Burkholderia*	5.21	Day 35

### Temporal variability of ocular surface microbiome in eyes treated with erythromycin antibiotic ointment

Two additional samples were obtained from treatment eyes following baseline (day 0) in order to observe the temporal stability of the ocular surface microbiome in healthy cats following topical antibiotic use. Sampling occurred after one week of antibiotic therapy was applied to the eye three times daily (day 7), and four weeks after discontinuing antibiotic therapy (day 35).

#### Species richness and diversity

**Fig 8:**
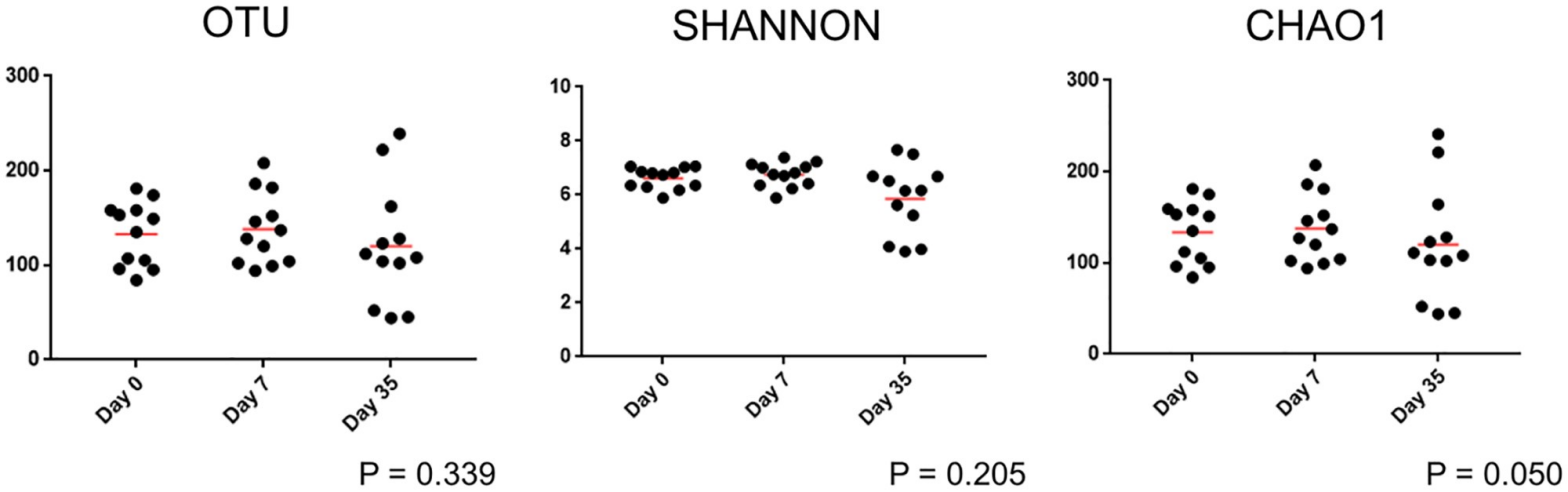
Scatter plots of 16S-rRNA gene sequences obtained from 12 treatment eyes of 12 healthy cats at 3 time points: Baseline (day 0), after one week of topical antibiotic therapy (day 7), four weeks after discontinued topical antibiotic therapy (day 35). There is no difference in alpha diversity in treated eyes over time (Freidman test and Dunn’s post-test).

Alpha diversity did not differ in treatment eyes based on the sampling time point (S4 Table and Fig 8).

#### Microbial community structure

**Fig 9:**
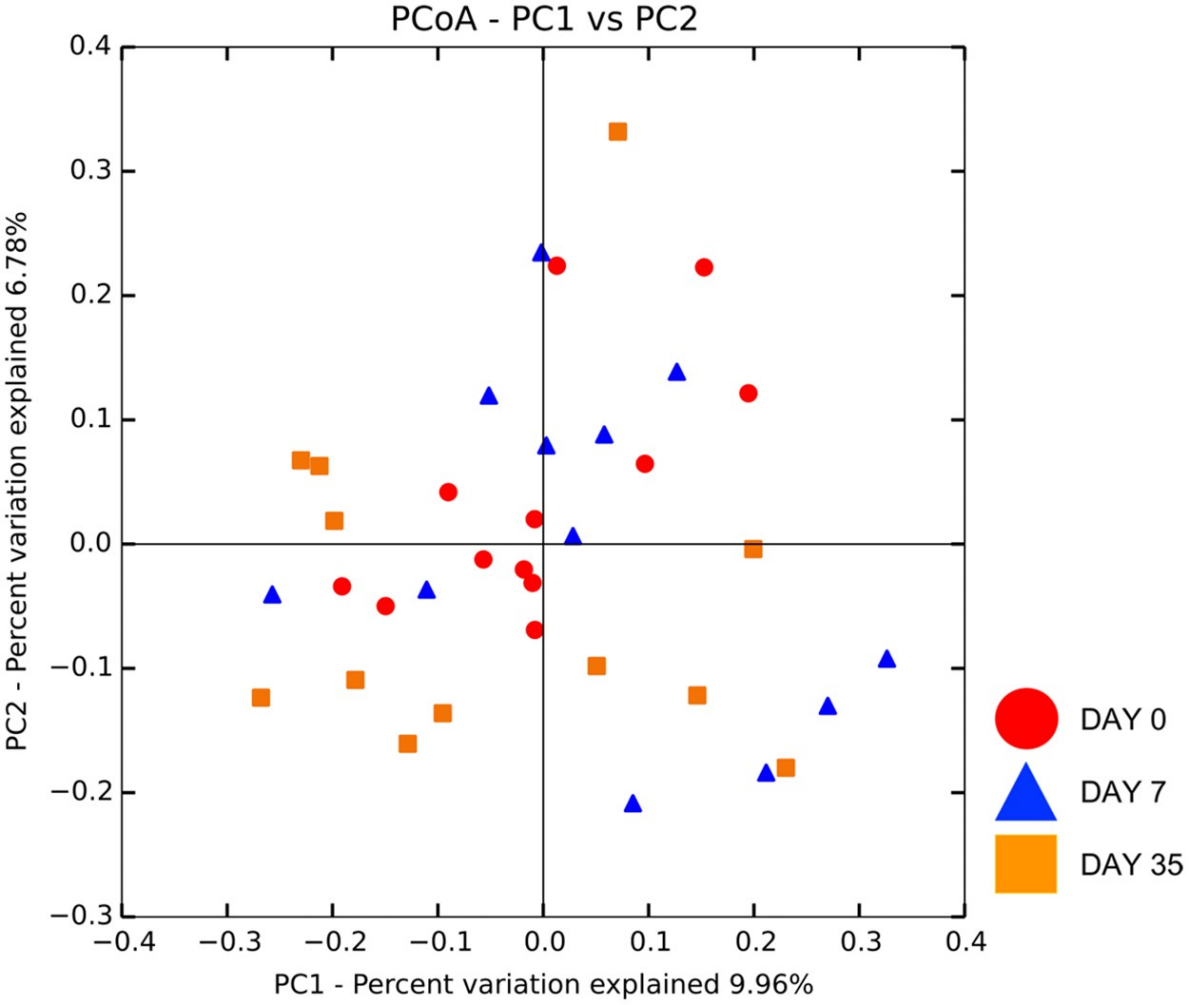
Principle coordinate analysis plot (PCoA) of unweighted UniFrac distance matrices of 12 treatment eyes from 12 healthy cats at three time points: Baseline (day 0), after one week of topical antibiotic therapy (day 7), four weeks after discontinued topical antibiotic therapy (day 35). Clustering was not observed indicating no difference in beta diversity in treatment eyes over time.

Beta diversity did not differ in treatment eyes sampled over time. This is apparent by the lack of clustering in the PCoA plot (Fig 9). No difference in microbial communities was detected with ANOSIM (unweighted UniFrac, R = 0.112, R = 0.187, R = 0.058 for day 0 vs. 7, day 0 vs. 35, and day 7 vs. 35, respectively, p > 0.05); (weighted UniFrac, R = 0.181, R = 0.359, R = 0.131 for day 0 vs. 7, day 0 vs. 35, and day 7 vs. 35, respectively, p > 0.05).

#### Microbial community composition

**Fig 10:**
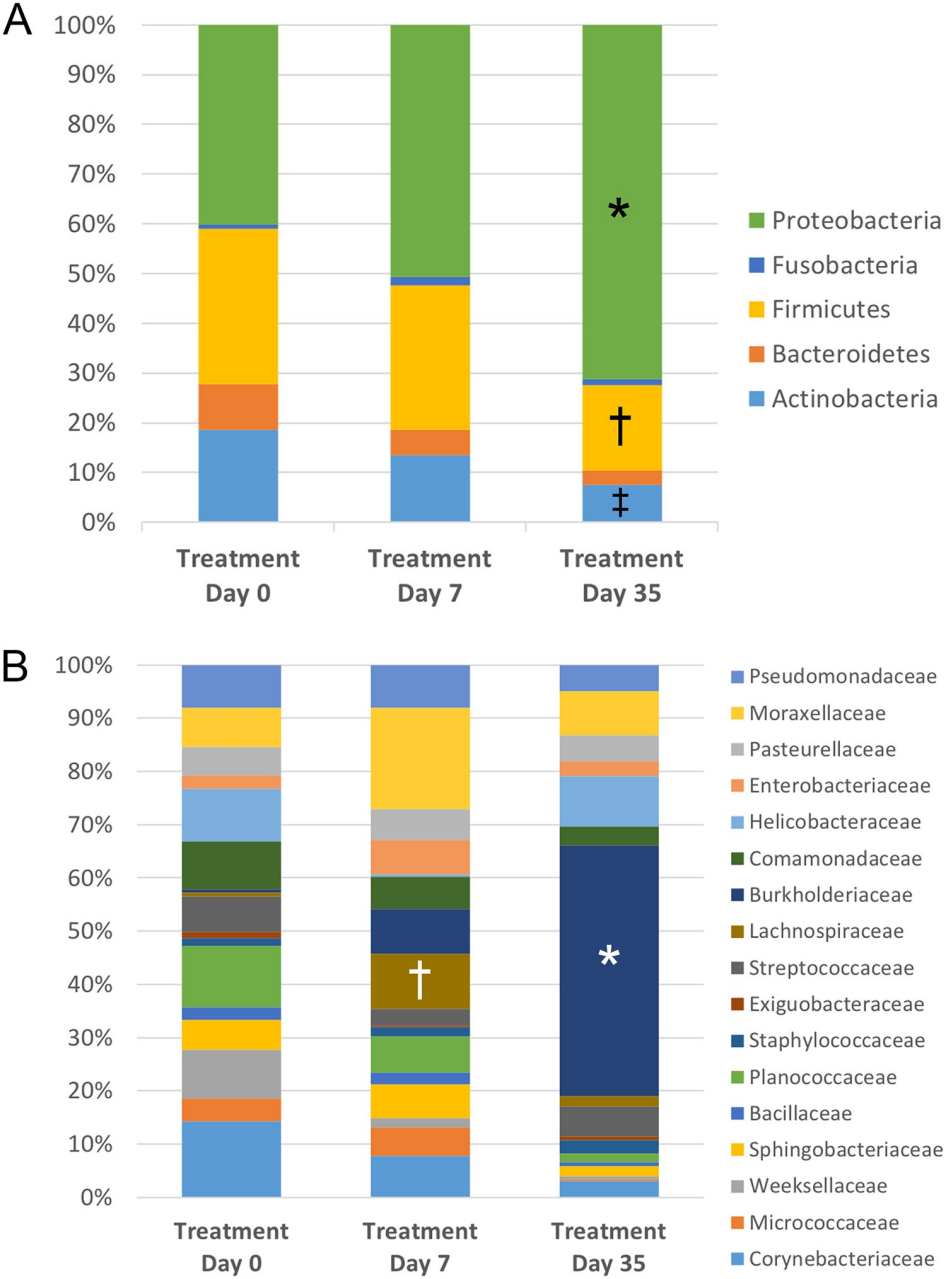
Temporal composition by bacterial phyla (A) and families (B) in treated eyes. Bars represent mean percentage of taxa present at ≥ 3% mean relative abundance. **(A)** Note the relative abundance of Proteobacteria (*) is increased on day 35, while Firmicutes (†) and Actinobacteria (‡) are decreased on day 35. **(B)** At the family level, the relative abundance of Burkholderiaceae (*) is increased on day 35 and Lachnospiraceae (†) is increased on day 7.

The relative abundance of bacteria from treatment eyes sampled over time is demonstrated in Fig 10. Seven taxa were differentially abundant on the ocular surface of treatment eyes over time (Table 4). At the phylum level, Proteobacteria were increased on day 35 compared to day 0 (p = 0.009, q = 0.037). Firmicutes were decreased on day 35 compared to day 0 and day 7 (p = 0.018, q = 0.037), and Actinobacteria were decreased on day 35 compared to day 0 (p = 0.017, q = 0.037). At the family level, Burkholderiaceae were increased on day 35 compared to day 0 (p = 0.001, q = 0.025). Lachnospiraceae were increased on day 7 compared to day 0 (p < 0.001, q = 0.018), and Microbacteriaceae were decreased on day 35 compared to day 0 (p = 0.002, q = 0.037). At the genus level, *Burkholderia* were enriched in treatment eyes on day 35 compared to day 0 (p < 0.001, q = 0.004).

**Table 4:** Temporal variation of bacterial taxa isolated from the ocular surface of treatment eyes of healthy cats at three time points. Median relative percentages and ranges of bacterial groups, annotated to level of phylum, family and genus, based on sequencing of 16S rRNA genes.

Taxa	Day 0		Day 7		Day 35			
Phylum Family *-Genus*	Median %	Range %	Median %	Range %	Median %	Range %	p-value*	q-value**
**Proteobacteria**	40.9^a^	13.2–65.9	46.2^a,b^	31.3–72.2	71.1^b^	44.7–94.5	**0.009**	**0.037**
Burkholderiaceae	0^a^	0–1.5	1^a,b^	0–43.1	17.4^b^	0.3–90.5	**0.001**	**0.025**
*-Burkholderia*	0^a^	0–1.4	0.5^a^	0–43.1	16.7^b^	0.3–90.5	**<0.001**	**0.004**
**Firmicutes**	28.4^a^	20.6–49.4	27.2^a^	13.7–44.2	11.7^b^	2.4–48.4	**0.018**	**0.037**
Lachnospiraceae	0^a^	0–2.5	3.2^b^	0–23.5	0.9^a,b^	0–6.9	**<0.001**	**0.018**
Streptococcaceae	4.2^a^	0.9–8.4	1.4^b^	0–0.8	0.9^b^	0–34	**0.006**	0.071
*-Streptococcus*	4.2^a^	0.3–8.4	1.4^a,b^	0–8.8	0.9^b^	0–34	**0.046**	0.134
Staphylococcaceae	5.8^a^	1–24	1.4^a,b^	0–27.5	0.5^b^	0–9.4	**0.028**	0.128
*-Staphylococcus*	5.7^a^	1–24	1.4^a,b^	0–27.5	0.5^b^	0–9.4	**0.028**	0.118
**Actinobacteria**	13.8^a^	9.2–43.3	9.7^a,b^	3.5–36.1	5.8^b^	0.1–20	**0.017**	**0.037**
Microbacteriaceae	1.3^a^	0–5.6	0.4^a,b^	0–5.7	0^b^	0–0.6	**0.002**	**0.037**
Corynebacteriaceae	8.3^a^	0–29.7	3.1^a,b^	0–16.2	1.0^b^	0–9.8	**0.014**	0.102
*-Corynebacterium*	8.3^a^	0–29.7	3.1^a,b^	0–16.2	1.0^b^	0–9.8	**0.014**	0.080
**Bacteroidetes**	8.1^a^	1.6–26.8	4.8^a,b^	0.1–16.3	1.8^b^	0–7.6	0.050	0.075

Median values not sharing a common superscript differ significantly (p < 0.05, Dunn’s multiple comparison post-test).

*: P-values based on the Friedman test

**: Q-values adjusted based on the Benjamini & Hochberg False discovery rate

Differences in the relative abundance of bacterial phyla, families, and genera over time was discovered with LEfSe (Table 5). As noted with the analysis of bacterial groups using univariate testing (Friedman and Dunn’s multiple comparison tests), *Burkholderia* spp. and their associated family, Burkholderiacaea, and phylum, Proteobacteria, were increased on day 35 among treatment eyes (Tables 4 and 5). Additionally, the phyla Firmicutes and Actinobacteria, as well as the family Microbacteriaceae, were most abundant on day 0, while Lachnospiraceae were enriched on day 7 (Tables 4 and 5).

**Table 5:** Linear discriminant analysis of bacterial taxa with LDA scores > 3.0 in treatment eyes and their associations with each time point.

Taxa	LDA	Time point
Phylum Family *-Genus*		
**Bacteroidetes**	4.45	Day 0
**Actinobacteria**	4.70	Day 0
**Firmicutes**	4.85	Day 0
**Proteobacteria**	5.17	Day 35
Actinomycetaceae	3.66	Day 0
Unclassified_Solibacterales	3.69	Day 0
Neisseriaceae	3.76	Day 0
Microbacteriaceae	4.10	Day 0
Streptococcaceae	4.22	Day 0
Weeksellaceae	4.39	Day 0
Staphylococcaceae	4.45	Day 0
Pseudonocardiaceae	3.66	Day 7
Campylobacteraceae	3.70	Day 7
Succinivibrionaceae	3.99	Day 7
Lachnospiraceae	4.46	Day 7
C111	3.63	Day 35
Burkholderiaceae	5.21	Day 35
-*Unclassified Pirellulaceae*	3.48	Day 0
*-Jeotgalicoccus*	3.54	Day 0
*-Haemophilus*	3.75	Day 0
-*Unclassified Solibacterales*	3.87	Day 0
-*Unclassified Comamonadaceae*	3.94	Day 0
*-Ralstonia*	3.98	Day 0
*-Streptococcus*	4.18	Day 0
*-Cloacibacterium*	4.39	Day 0
*-Staphylococcus*	4.44	Day 0
*-Campylobacter*	3.62	Day 7
*-Paracoccus*	3.63	Day 7
*-Parabacteroides*	3.68	Day 7
*-Enhydrobacter*	3.72	Day 7
*-Faecalibacterium*	3.73	Day 7
*-Pseudonocardia*	3.81	Day 7
*-Blautia*	4.03	Day 7
-*Unclassified C111*	3.73	Day 35
-*Unclassified Clostridiaceae*	4.20	Day 35
*-Burkholderia*	5.24	Day 35

A direct comparison of taxa between control eyes and treatment eyes on day 7 and day 35 did not reveal a difference in either alpha or beta diversity (S5 Table) (unweighted UniFrac, R = -0.06, R = -0.07, for day 7 and day 35, respectively, p > 0.05); (weighted UniFrac, R = 0.05, R = -0.06 for day 7 and 35, respectively, p > 0.05). The feline ocular surface microbiome displays an overall lack of clustering when comparing treatment and control eyes throughout the experimental design (S1 Fig).

## Discussion

Advancements in sequencing technologies have enhanced our understanding of microbial composition and diversity in humans and animals. The present study reveals the feline ocular surface consists of a more complex and diverse bacterial community than previously detected using standard culture-based techniques. Five bacterial phyla and 34 bacterial families were detected at >1% relative abundance (Table 1). Throughout all samples, an average of 353 observed species were detected on the feline eye.

The most common phyla and their relative proportions colonizing the feline ocular surface, Proteobacteria (42.4%), Firmicutes (30.0%), Actinobacteria (15.6%), and Bacteroidetes (8.1%), are similar to investigations of the human [38, 39, 42] and equine [44] ocular surface microbiome. Preliminary studies describing the ocular surface microbiome of cats and dogs utilizing NGS also identified Proteobacteria and Firmicutes as the two most common bacterial phyla, though at different proportions [41, 45]. For example, Firmicutes (43%) had the highest relative abundance followed by Proteobacteria (30%) across all samples from 14 healthy cats [41]. Even though the current study appears analogous to previous investigations, the comparison of microbiome studies utilizing NGS should be performed with discretion as a multitude of variations exist among methodologies for DNA extraction, sequencing, and analysis.

The most relatively abundant bacterial families sequenced in 96–100% of feline eyes sampled were Corynebacteriaceae (7.8%), Helicobacteraceae (7.5%), Moraxellaceae (6.1%), Comamonadaceae (5.6%), Pseudomonadaceae (5.4%), Staphylococcaceae (5.3%), and Streptococcaceae (4.4%) (Table 1). The majority of the most relatively abundant microorganisms isolated were gram-negative (24.6%; Helicobacteraceae, Moraxellaceae, Comamonadaceae, Pseudomonadaceae) compared to gram-positive (17.5%; Corynebacteriaceae, Staphylococcaceae, Streptococcaceae). This finding challenges the previous notion reported in the culture-based literature that the feline ocular surface is dominated by gram-positive bacteria [1–3, 18–22].

By utilizing NGS, this study identified several taxa previously unassociated with the feline ocular surface from culture-based reports, likely due to their lack of cultivability. This includes families from the phyla Proteobacteria (Helicobacteraceae, Comamonadaceae, Halomonadaceae, Xanthomonadaceae, Rhodobacteraceae, Oxalobacteraceae, Sphingomonadaceae), Firmicutes (Aerococcaceae, Ruminococcaceae, Tissierellaceae, Lactobacillaceae, Exiguobacteraceae, Planococcaceae, Gemellaceae, Lachnospiraceae, Erysipelotrichaceae, Clostridiaceae), Actinobacteria (Bifidobacteriaceae), and Bacteroidetes (Weeksellaceae, Bacteroidaceae, Porphyromonadaceae) (Table 1). Of the aforementioned families, Aerococcaceae, Ruminococcaceae, Planococcaceae, and Lachnospiraceae were sequenced from the conjunctiva of cats with and without feline immunodeficiency virus (FIV) in a preliminary study using NGS [41]. A complete understanding of the impact of these recently identified organisms on the health and disease status of the feline ocular surface remains to be elucidated.

There was no difference in beta diversity among control eyes when sampled at three separate time points: day 0, day 7, and day 35. Likewise, a significant difference was not detected in the following alpha diversity matrices in control eyes over time: observed OTUs and Chao1. This finding suggests the ocular surface microbiome maintains temporal stability with regard to species richness and community structure. Shannon diversity was significantly lower in control eyes on day 35 compared to day 7 (Fig 5). A decrease in Shannon diversity indicates a decrease in the abundance and evenness of species. This finding likely corresponds to a change in the relative abundance of some bacterial taxa over time. Univariate and linear discriminant analyses revealed that Proteobacteria (at the phylum level) and *Burkholderia* spp. (at the genus level) were significantly increased on day 35 compared to day 0 and day 7 (Tables 2 and 3).

The feline ocular surface is likely composed of both a core and transient microbiome. Although there was some temporal variability in community composition detected in control eyes, the vast majority of identified bacterial taxa were present in most eyes at every time point, with no significant change in their relative abundance over time. We speculate many of the bacterial families listed in Figs 7B and 10B belong to the core microbiome; however, additional cross-sectional and longitudinal studies are needed to support our findings. Concurrently, there was individual variation in the relative abundance of taxa both between eyes and between cats at baseline. This finding is not unique to the ocular surface, as it is generally recognized that a high degree of interindividual variability exists within human and animal microbiomes, and this is likely attributed to environmental factors and host genetics [53].

Currently, there is debate within the physician ophthalmology literature on whether a true core ocular surface microbiome exists, or if it is merely composed of a transient community of microbes from the surrounding environment [10, 13, 37–40, 43, 54]. Compelling arguments can be crafted with supporting evidence that the ocular surface is unfavorable for the establishment of a core microbiome due to its unique innate immune defenses [2, 4, 19 20], thus making a transient population of microorganisms a more likely possibility. Literature has also shown that there is a lower microbial biomass and diversity on the ocular surface compared to other organ systems such as the gastrointestinal tract, oral cavity, nasal cavity, and skin [10, 37, 38, 42, 43]. However, the ocular surface has its own unique and distinctive bacterial microbiome when compared to these other regions of the body, and it appears to be relatively stable over time [38, 43]. With this knowledge, it is conceivable to speculate that the ocular surface microbiome is composed of both a stable commensal core and a transient collection of environmental microbes that encounter the eye.

There were no differences in either alpha or beta diversity among treatment eyes when sampled at baseline (day 0), after one week of topical ophthalmic antibiotic therapy with erythromycin (day 7), and four weeks after discontinuing antibiotic therapy (day 35). As reported with the control eyes, there were statistically significant differences in the relative abundance of some bacterial taxa over time (Tables 4 and 5). Both univariate and linear discriminant analyses revealed *Burkholderia* spp. and their associated family, Burkholderiaceae, and phylum, Proteobacteria, were increased among treatment eyes on day 35 compared to day 0. Additionally, the phyla Firmicutes and Actinobacteria, as well as the family Microbacteriaceae, were most abundant on day 0, while Lachnospiraceae were enriched on day 7 (Tables 4 and 5). A similar trend was noted among control eyes. These findings suggest a short-term course of broad-spectrum topical antibiotics does not alter the feline ocular surface microbiome with regard to species richness, community structure, and global community composition. Erythromycin is a macrolide antibiotic that is primarily effective against gram-positive bacteria. Minor shifts in abundance of some bacterial taxa over time, such as with *Burkholderia*, a gram-negative organism, were similar in both treatment and control eyes and likely more indicative of transient changes that occur due to external factors in an open system as opposed to the influence of antibiotic therapy. As the bioavailability of topically applied medications is extremely low (<5%), systemic absorption of erythromycin was unlikely to reach therapeutic dosages to the ocular surface of the contralateral eye [55].

The genus *Burkholderia* consists of rod-shaped bacteria and has approximately 100 validated species with a majority being ubiquitous in the environment as soil inhabitants [56]. Two well-known species of *Burkholderia*, *B*. *pseudomallei* and *B*. *mallei*, are associated with zoonotic diseases such as glanders and melioidosis, respectively. Currently, there is no evidence of ocular disease linked to *Burkholderia* species in veterinary medicine. Within the physician ophthalmology literature, *Burkholderia* was suspected to cause endophthalmitis and keratitis in a small number of case reports [57–59]. The temporal increase in the relative abundance of *Burkholderia* spp. in this population of cats remains of unknown consequence and is likely an incidental and transient environmental fluctuation. Another possible consideration for the presence of *Burkholderia* in relatively low biomass samples could be due to contamination from the DNA extraction kit or PCR and amplification reagents [60]. While there is no evidence of *Burkholderia* causing ocular disease in felines, further investigation into the ocular pathogenicity of this genus of bacteria is warranted.

Although the ocular surface microbiome was not significantly impacted by a short-course of topical antibiotic therapy in the current study, more frequent and chronic application of ophthalmic antibiotics could have more profound effects and potentially facilitate the emergence of resistant strains [27, 61]. Infectious conjunctivitis occurs frequently in cats and this condition is often treated with topical antibiotics for a prolonged period of three weeks or greater [1,18,22]. Additionally, many cats will receive several treatment trials in their lifetime as recurrence is common. Etiologically, feline herpes virus-1, *Chlamydia* spp. and *Mycoplasma* spp. are commonly associated with feline conjunctivitis [1,18,22]. Erythromycin accumulates intracellularly allowing for effective treatment against conjunctival *Chlamydia* and *Mycoplasma* infections in cats. In the present study, *Chlamydia* spp. were not isolated from the eyes of 12 healthy cats at any timepoint; however, *Mycloplasma* spp. were identified in 1/24 eyes in the control group at baseline at less than 1% relative abundance (0.002%). Previous culture-based studies have isolated *Mycoplasma* from both diseased and healthy feline eyes [18]. Therefore, its exact role in causing conjunctivitis is unclear. Clinical signs of ocular disease were not present in any cat throughout the study period. Future studies are necessary to evaluate the effects of chronic antimicrobial use in the face of disease on the feline ocular surface microbiome utilizing NGS. Investigating the composition of the ocular surface microbiome and the impact of prolonged antibiotic usage will not only aid clinically with antibiotic stewardship, but also help combat the crisis of antibiotic resistance that plaques physician and veterinary medicine alike.

The homogeneity of the study population provided an ideal circumstance to study the ocular surface microbiome. All 12 cats were adult females, housed in the same building, fed the same diet, and exposed to the same exogenous factors (enrichment toys, caretakers, and research personnel). This provided a highly controlled environment in which to evaluate the ocular surface microbiome. However, there are limitations to studying a homogenous population, as our findings may not represent the general feline population. It is possible that more diverse feline populations with different ages, sex, and environmental factors such as geography, housing, and diet, may demonstrate marked differences in their resident microbial populations. Additionally, cats in this study were housed in groups of 2–4 within the same enclosures, and such close social interactions may affect the composition of the ocular surface microbiome. The feline species is known for its superior ability to groom and allogrooming/allorubbing can be a social activity shared between felids who live in close proximity to reinforce bonds, establish hierarchy, and develop companionships [62]. This may cause them to share a larger percentage of their individual microbiome composition compared to cats in more solitary living conditions. Measures were not taken to prevent grooming in this study as this is an expected behavior and known environmental factor that occurs in their natural habitat.

Literature on the feline ocular microbiome is scarce at this time with only one other study utilizing NGS to the authors’ knowledge. Therefore, interpretation of the significance of the ocular surface microbiome composition, its stability, and response to antibiotic therapy is challenging. Larger scale evaluations from more heterogeneous populations of cats are warranted to limit bias and expound upon the data presented within this study. Further evaluation of the effects of chronic antibiotic use, as well as other exogenous and endogenous factors may allow for a more comprehensive and clinically relevant understanding of the ocular surface microbiome.

Additional limitations of this study are those inherent to NGS. For example, the evaluation of relative abundance does not represent absolute quantities of the microbial populations present [53]. Quantitative PCR of specific organisms is required to determine absolute abundance of a known sequence within a sample. In addition, NGS does not determine the viability of organisms present in a sample. Therefore, organisms that are detected via NGS may represent more than just a living community of organisms on the ocular surface but also a collection of nonviable organisms that have fallen prey to the host’s immune defenses [37]. Given the relatively low biomass environment of the ocular surface, contaminating DNA from laboratory reagents may also impact the results obtained [60]. Even with such limitations, NGS provides a plethora of useful knowledge that, along with future investigations, will enhance our understanding of the ocular surface microbiome and its role in health and disease.

## Conclusion

This is the first report to investigate the temporal stability of the feline ocular surface microbiome both in untreated eyes and following topical antibiotic therapy. Using molecular-based techniques, a diverse, species-rich bacterial community was shown to inhabit the healthy feline ocular surface. In contrast to culture-based studies, all eyes demonstrated the presence of bacterial microbes, many of which were gram-negative and previously unassociated with the feline eye. A stable bacterial microbiome was identified and discovered to remain consistent with regard to species richness and community structure both over time and following one week of antibiotic therapy. However, significant and similar changes in the abundance of some bacterial taxa over time in both treatment and control eyes indicate the open nature of the ocular surface microbiome is likely influenced by external environmental factors. Further studies are warranted to elucidate if the ocular surface microbiome is altered in the face of disease and chronic topical antibiotic use.

## Supporting information

S1 Table Quantification of nucleic acid (ng/μl) extracted from conjunctival swabs of healthy cats. (DOCX)

S2 Table Summary of alpha diversity indices at a depth of 15,999 sequences per sample for control and treatment eyes at baseline. (DOCX)

S3 Table Summary of alpha diversity indices at a depth of 15,999 sequences per sample for control eyes over time. (DOCX)

S4 Table Summary of alpha diversity indices at a depth of 15,999 sequences per sample for treatment eyes over time. (DOCX)

S5 Table Summary of alpha diversity indices at a depth of 15,999 sequences per sample comparing control eyes and treatment eyes at day 7 and day 35. (DOCX)

S1 Fig Principle coordinate analysis plot (PCoA) of unweighted UniFrac distance matrices of 12 treatment eyes and 12 control eyes from 24 healthy cats at three time points: Baseline (day 0), after one week of topical antibiotic therapy (day 7), four weeks after discontinued topical antibiotic therapy (day 35). No clustering was observed indicating there was no difference in beta diversity in control or treatment eyes over time.
(TIF)
