# Supplementary material for: Evaluation of the bacterial ocular surface microbiome in clinically normal cats before and after treatment with topical erythromycin
Source: PLoS One. 2019 Oct 11;14(10):e0223859. doi: 10.1371/journal.pone.0223859 (PMC6788832; doi:10.1371/journal.pone.0223859)
Supplement: S1 Table — (DOCX) [file pone.0223859.s001.docx]

**S1 Table. Quantification of nucleic acid (ng/µl) extracted from conjunctival swabs of healthy cats.**

|  | **Day 0** | **Day 7** | **Day 35** |
| --- | --- | --- | --- |
| **Control Eyes** | 11 ± 6 | 12 ± 5 | 11 ± 7 |
| **Treatment Eyes** | 14 ± 8 | 11 ± 4 | 12 ± 6 |
| **Unused Swab (Negative Control)** | 3 | 2 | 3 |

Values from control and treatment eyes represent averages with standard deviations.
