# Supplementary material for: Evaluation of the bacterial ocular surface microbiome in clinically normal cats before and after treatment with topical erythromycin
Source: PLoS One. 2019 Oct 11;14(10):e0223859. doi: 10.1371/journal.pone.0223859 (PMC6788832; doi:10.1371/journal.pone.0223859)
Supplement: S2 Table — (DOCX) [file pone.0223859.s002.docx]

**S2 Table. Summary of alpha diversity indices at a depth of 15,999 sequences per sample for control and treatment eyes at baseline.**

|  | **Control Eyes** | **Treatment Eyes** | ***P-value** |
| --- | --- | --- | --- |
| **Observed OTUs** | 133 ± 34 | 128 ± 47 | 0.854 |
| **Shannon** | 7 ± 0.5 | 7 ± 0.4 | 0.970 |
| **Chao1** | 128 ± 47 | 133 ± 34 | 0.424 |

Values represent averages with standard deviations. *P-values determined by Wilcoxon matched-pairs signed-ranks test with significance level < 0.05.
