# Supplementary material for: Evaluation of the bacterial ocular surface microbiome in clinically normal cats before and after treatment with topical erythromycin
Source: PLoS One. 2019 Oct 11;14(10):e0223859. doi: 10.1371/journal.pone.0223859 (PMC6788832; doi:10.1371/journal.pone.0223859)
Supplement: S3 Table — (DOCX) [file pone.0223859.s003.docx]

**S3 Table. Summary of alpha diversity indices at a depth of 15,999 sequences per sample for control eyes over time.**

| **Control Eyes** | **Day 0**  **(Baseline)** | **Day 7** | **Day 35** | ***P-value** |
| --- | --- | --- | --- | --- |
| **Observed OTUs** | 128 ± 47 | 156 ± 51 | 106 ± 33 | 0.076 |
| **Shannon** | 7 ± 0.6 | 7 ± 0.5^*^ | 6 ± 1^*^ | 0.013 |
| **Chao1** | 128 ± 47 | 156 ± 51 | 107 ± 33 | 0.076 |

Values represent averages with standard deviations. *P-values determined by Freidman test and Dunn’s post-test with significance level < 0.05.
