# Supplementary material for: Evaluation of the bacterial ocular surface microbiome in clinically normal cats before and after treatment with topical erythromycin
Source: PLoS One. 2019 Oct 11;14(10):e0223859. doi: 10.1371/journal.pone.0223859 (PMC6788832; doi:10.1371/journal.pone.0223859)
Supplement: S4 Table — (DOCX) [file pone.0223859.s004.docx]

**S4 Table. Summary of alpha diversity indices at a depth of 15,999 sequences per sample for treatment eyes over time.**

| **Treatment Eyes** | **Day 0**  **(Baseline)** | **Day 7** | **Day 35** | ***P-value** |
| --- | --- | --- | --- | --- |
| **Observed OTUs** | 133 ± 33 | 138 ± 38 | 120 ± 63 | 0.339 |
| **Shannon** | 7 ± 0.6 | 7 ± 0.5 | 6 ± 1 | 0.205 |
| **Chao1** | 134 ± 34 | 138 ± 38 | 120 ± 63 | 0.050 |

Values represent averages with standard deviations. *P-values determined by Freidman test and Dunn’s post-test with significance level < 0.05.
