# Supplementary material for: Evaluation of the bacterial ocular surface microbiome in clinically normal cats before and after treatment with topical erythromycin
Source: PLoS One. 2019 Oct 11;14(10):e0223859. doi: 10.1371/journal.pone.0223859 (PMC6788832; doi:10.1371/journal.pone.0223859)
Supplement: S5 Table — (DOCX) [file pone.0223859.s005.docx]

**S5 Table. Summary of alpha diversity indices at a depth of 15,999 sequences per sample comparing control eyes and treatment eyes at day 7 and day 35.**

|  | **Control Eyes** | **Treatment Eyes** | ***P-value** |
| --- | --- | --- | --- |
| **Day 7** | | | |
| **Observed OTUs** | 156 ± 51 | 138 ± 38 | 0.453 |
| **Shannon** | 7 ± 0.5 | 7 ± 0.5 | 0.453 |
| **Chao1** | 156 ± 51 | 138 ± 38 | 0.470 |
| **Day 35** | | | |
| **Observed OTUs** | 106 ± 33 | 120 ± 63 | 0.751 |
| **Shannon** | 6 ± 1 | 6 ± 1 | 0.817 |
| **Chao1** | 107 ± 33 | 120 ± 63 | 0.729 |

Values represent averages with standard deviations. *P-values determined by Wilcoxon matched-pairs signed-ranks test with significance level < 0.05.
