# Supplementary figures and images for: Evaluation of the bacterial ocular surface microbiome in clinically normal cats before and after treatment with topical erythromycin
Source: PLoS One. 2019 Oct 11;14(10):e0223859. doi: 10.1371/journal.pone.0223859 (PMC6788832; doi:10.1371/journal.pone.0223859)

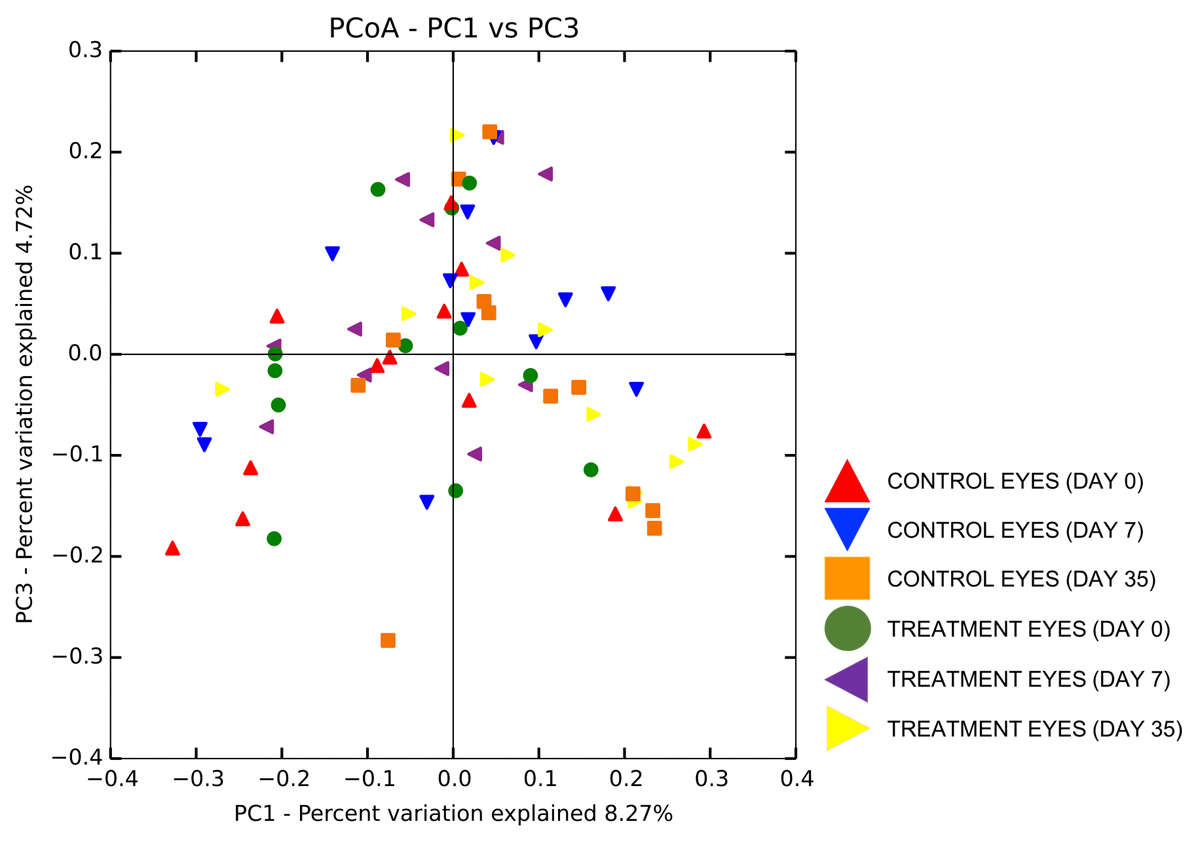

Supplement: S1 Fig — No clustering was observed indicating there was no difference in beta diversity in control or treatment eyes over time. (TIF) [file pone.0223859.s006.tif]
